# Papillary Growths of the Leg Preceding Cancer

**Published:** 1882-07

**Authors:** 


### PAPILLARY GROWTHS OF THE LEG PRECEDING CANCER.

We had some time ago, through the kindness of Dr. Stephen Mackenzie, several opportunities of seeing a man who has some very peculiar growths on the lower part of one leg. There are several patches of a papillary growth close together, and almost confluent, a quarter of an inch or more in height. On their surface the papillary outgrowths are covered by a dirty scab, but if we detach or break this you can easily separate the wart-like growths from each other and put them apart in long rows, like standing corn. They do not readily bleed, and the skin from which they grow is somewhat thickened. The main patch is over the shin, in front of the lower third of the leg, and is nearly as large as the palm of the hand, but near it are several much smaller ones. The first stage of the condition is a thickened and rough papule, not unlike a spot of psoriasis, but with more evidence of growth. Our patient is a man of near fifty, and he has had the condition for two or three years. It gives him no trouble. He has no enlarged glands.

I do not think that there can be much hesitation in considering that the disease is a variety of papilloma which, if not actually cancerous at present, is in a fair way to become so. At any time we might find the glands enlarged, or that the patch had begun to ulcerate. It is mainly in the absence of any ulceration that the picture of epithelioma is as yet incomplete. There is no telling how long or how short may be the period during which its appearance will be delayed. I have urged the man to at once submit to radical treatment; that is, to have the growths freely destroyed by chloride-of-zinc paste.

Dr. Mackenzie's patient shows an extreme condition of what, in lesser forms, I have often seen before. An old man, aged seventy-four, came to us at the Hospital for Skin-diseases last week who had exactly the same thing, but in smaller areas and with less elevation. They had been present two years, and excepting that they itched excessively, gave him no tiouble. In him one of the patches was at the back of the leg, most of them, however, being in the usual position—over the shin. I will ask you to note in connection with this fact that the patches are almost always multiple—a chief one and several smaller near to it. Although I ask you to believe that this disease is really a close ally of cancer, yet I do not know that I can quote any complete case in which I have watched patches which began as I have described and progressed to an undoubtedly cancerous termination ; but I have seen several that were cancerous in which there was good reason to believe that the beginning had been like them. It is only in the legs of aged people that we see such growths, and the liability to them seems to begin at the cancerous time of life. Often some local irritation, eczema, etc., precedes their formation. Not unfrequently, during periods of some years, the patches remain without warty or papillary outgrowth, simply hard, rough patches, well circumscribed, and showing on the surface the little points or buds from which future growth is to take place. In this stage they resemble condylomata, excepting in their hardness and roughness. Often, however, they are not round, but in long streaks and very irregular. Probably they are for the skin of the leg what the rodent cancer is for the upper part of the face—the form of malignant action which it is most prone to take on. I have not often seen them on other parts of the body.—*British Med. Journal.*

